# Relationship Factors in Internet-Delivered Psychological Interventions for Veterans Experiencing Postpartum Depression: Qualitative Analysis

**DOI:** 10.2196/46061

**Published:** 2023-08-15

**Authors:** Cara L Solness, Paul J Holdefer, Ti Hsu, Emily B K Thomas, Michael W O'Hara

**Affiliations:** 1 Department of Psychiatry, School of Medicine University of Colorado Aurora, CO United States; 2 Department of Psychological and Brain Sciences University of Iowa Iowa City, IA United States

**Keywords:** internet-delivered treatment, postpartum depression, therapeutic alliance, qualitative methodology, veteran

## Abstract

**Background:**

Internet-delivered psychological interventions (IPIs) have been shown to be effective for a variety of psychological concerns, including postpartum depression. Human-supported programs produce better adherence and larger effect sizes than unsupported programs; however, what it is about support that affects outcomes is not well understood. Therapeutic alliance is one possibility that has been found to contribute to outcomes; however, the specific mechanism is not well understood. Participant perspectives and qualitative methodology are nearly absent from the IPI alliance research and may help provide new directions.

**Objective:**

In this study, we aimed to provide participant perspectives on engagement with an IPI for postpartum depression to help inform alliance research, development of new IPIs, and inform resource allocation.

**Methods:**

A qualitative methodology was used to explore participant perspectives of veteran women’s engagement with the MomMoodBooster program, a human-supported internet-delivered intervention for postpartum depression. Participants were asked 4 open-ended questions with the 3-month postintervention survey, “In what ways did you find the MomMoodBooster most helpful?” “How do you think the MomMoodBooster could have been improved?” “In what ways did you find the personal coach calls to be helpful?” and “How do you think the personal coach calls could have been improved?”

**Results:**

Data were collected from 184 participants who responded to at least 1 of the open-ended questions. These were analyzed using thematic analysis and a process of reaching a consensus among coders. The results suggest that not only the engagement with the support person is perceived as a significant contributor to participant experiences while using the MomMoodBooster content but also the relationship factors are particularly meaningful. The results provide insights into the specific qualities of the support person that were perceived as most impactful, such as warmth, empathy and genuineness, and feeling normalized and supported. In addition, the results provide insight into the specific change processes that can be targeted through support interactions, such as encouraging self-reflection and self-care and challenging negative thinking.

**Conclusions:**

These data emphasize the importance of relationship factors between support persons and an IPI program for postpartum depression. The findings suggest that focusing on specific aspects of the alliance and the therapeutic relationship could yield fruitful directions for the training of support personnel and for future alliance-based research of internet-delivered treatments.

## Introduction

### Background

Internet-delivered psychological interventions (IPIs; see the *Terminology* section) for postpartum depression are an important part of expanding the reach of specialized mental health services and thus reducing barriers to treatment. Less than half of those affected by mental health concerns seek or receive evidence-based treatments owing to costs, limited access, stigma, and preference to self-manage [1]. IPIs address these issues and offer anonymity, flexibility, and cost-effectiveness [2]. Several meta-analyses have found IPIs to be efficacious for treating depression [3-6] and specifically for postpartum depression [7].

Previous research has demonstrated that human-supported IPIs are more effective than unsupported ones [7-9]. However, what it is about support that makes treatment more effective is not well understood [10]. Research incorporating the perspectives of those who use IPIs may provide insight into these mechanisms. Given the importance of resource allocation, more work is needed to understand the specific mechanisms that account for the effect of support, and qualitative methods are central to this endeavor [10,11].

Therapeutic alliance is one possible explanation for the effects of support. Therapeutic alliance, as most commonly defined in the IPI literature, is based on the definition by Bordin [12,13] who suggests the conceptualization of a positive emotional bond between a therapist and a client, including mutual agreement on the goals and tasks of the treatment. In face-to-face (FTF) therapy, the therapeutic alliance is widely regarded as an important pantheoretical ingredient and accounts for approximately 8% of the variability in outcomes in FTF therapy [14,15]. Meta-analyses examining the association between therapeutic alliance and outcomes for IPIs indicate that there is a small and significant correlation between therapeutic alliance and various treatment outcomes (eg, anxiety disorders, anxiety related to preterm labor, obsessive compulsive disorder, depression, posttraumatic stress disorder, psychosis, schizophrenia, and tinnitus) [16,17]. Furthermore, this association does not appear to depend on the frequency of contact or on the mode of contact with the therapist or on the availability of self-help content [16]. The association between therapeutic alliance and treatment outcomes also does not depend on the amount of contact, the occurrence of FTF contact, or the timing of the alliance assessment [17].

Within the growing body of literature examining the therapeutic alliance in IPIs in general [16,18] and those examining depression outcomes specifically [10], some studies explore the alliance between participants and the computerized content [19], whereas others examine the alliance between participants and the support personnel [20,21]. In general, ratings of alliance in IPIs have been found to be similar to those in FTF therapies, and these ratings are positively associated with outcomes, although they do not reach statistical significance in some studies [22,23]. The systematic review of the alliance literature for guided IPIs by Pihlaja et al [23] examined only studies for which support was delivered via email and similarly found that the strength of the alliance was predictive of outcomes in some studies, whereas in others, the direction was positive but did not reach statistical significance. Other authors have concluded that the IPI alliance literature has provided mixed results [18]. However, more recent work on the alliance in IPIs such as the meta-analysis by Probst et al [17] found high homogeneity of effect sizes among their included studies. To summarize, although previous research on the role of the therapeutic alliance in IPIs provided some support but with mixed results, newer meta-analyses are less ambiguous about its contribution to various mental health outcomes [16,17].

Out of various IPIs, internet-delivered cognitive behavioral therapy (iCBT) has emerged as the most common and most studied intervention [18,24]. Previous research on iCBT has also examined the contribution of therapeutic alliance in this specific approach. For instance, a recent meta-analysis of phone-supported iCBT found a large effect size between alliance and outcomes [16], with the strength of the association only slightly smaller than that of FTF cognitive behavioral therapy (CBT; *r*=0.20 and *r*=0.29, respectively). The narrative review of the IPI alliance literature by Berger [18] concludes that guidance in iCBT plays a crucial role in outcomes and that the specific aspects of the alliance and therapist factors that facilitate positive outcomes are not yet well understood in the IPI literature.

Previous studies have examined therapist factors that contribute to the alliance. For example, Hilsenroth et al [25] found positive associations between the alliance and therapist characteristics such as openness, warmth, confidence, flexibility, honesty, tension, use of self-disclosure and negative associations with the alliance for characteristics such as rigidity, defensiveness, self-focus, and others in FTF therapy. The meta-analysis by Nienhuis et al [14] examined the associations between therapist empathy and genuineness and therapeutic alliance in individual FTF therapy and found a moderate association. When examining email-supported iCBT for generalized anxiety disorder, Paxling et al [26] identified the most common therapist behaviors in written correspondence as deadline flexibility, task reinforcement, alliance bolstering, task prompting, psychoeducation, self-disclosure, self-efficacy shaping, and empathetic utterances. Similarly, Holländare et al [27] examined common behaviors within email content between therapists and patients and found positive associations between therapists’ behaviors, such as encouraging, guiding, and urging the patients and affirming their thoughts, and outcomes in iCBT for depression. Similar work on factors affecting alliance has not yet been completed specifically for telephone-supported IPIs. Furthermore, it is not possible to establish best practices for the provision of differently delivered support without first understanding the specific mechanisms identified by those using these interventions. As such, there is much to be gained from participant perspectives of their experiences with a telephone-supported IPI.

Questions related to the broad conceptualization of the alliance for IPIs and the extent to which traditional measures accurately represent what is occurring in IPIs are just beginning to be asked. For example, Askjer and Mathiasen [28] raised the question as to how to conceptualize the therapeutic alliance in blended treatments and suggested that an aggregated alliance (ie, to the program and to the support person or therapist) may be a more accurate representation of alliance in the blended format. Blended formats consist of self-directed content interspersed with FTF sessions. It may be that IPIs with regular, synchronous, phone-based support are more similar to blended treatments than asynchronous, text-supported IPIs are, which raises the question of how alliance is being measured for each IPI format. To address this question, Linder et al [29] compared telephone- and email-supported interventions for depression and found no difference, but their study was limited by a small sample size. The meta-analysis by Kaiser et al [16] provides additional information but leaves some questions that may be addressed via participant perspectives. For example, their meta-analysis found no impact on the association between alliance and outcomes for the frequency of contact with a therapist, mode of contact, or availability of self-help content. However, no information was provided about approach to the contact (ie, accountability based, support based, review based, education based, etc), which leaves open the possibility that categorical coding of support type may not be sufficient for detecting differences. More work is needed in this area.

Current literature examining the alliance commonly uses the Working Alliance Inventory (WAI) [30], which was developed for FTF therapy. Recent literature suggests that the task and goal subscales of the therapeutic alliance are more important than the therapeutic bond subscale in supported IPIs [10,17,18,31]. However, the literature has yet to examine the psychometric properties of the WAI, specifically with IPI users. Askjer and Mathiasen [28] found that clients and therapists rated the goal dimension higher than task and bond and that therapists rated bond higher than clients did; however, the literature does not address whether there is a difference in these subscales depending on the format of support. For example, it may be that IPI users perceive the therapeutic bond differently between these 2 types of support models. The question becomes whether the WAI is sensitive enough to be able to make this kind of distinction and whether it is in fact measuring the same construct in each case. In order to begin to address questions such as these, participant perceptions and experiences of IPIs, the support relationship, and the relative importance of each will be helpful toward the endeavor of conceptualizing alliance within synchronous, telephone-supported IPIs.

### Objectives of This Study

To address the gap in the literature of participant experiences with IPIs and the conceptualization of alliance in IPIs, this study examined participant-identified program and coach contributions to their experiences of using a human-supported iCBT delivered to veteran women with postpartum depression. The MomMoodBooster (MMB) program is an empirically supported iCBT intervention [32-34] administered to veteran women in the postpartum period [35] for which support was provided synchronously by telephone. The program from which these data were collected consisted of 6 self-directed, postpartum-specific, web-based CBT–based modules and 6 weekly concurrent phone coaching calls. A previous study described quantitative metrics of engagement and outcomes and provided further information regarding the methods of the intervention study [35]. On average, veterans engaged in 3.35 coaching calls and spent an average of 49.19 minutes on the phone with their coach over the course of their engagement with the program. Effect sizes for depressive symptoms, behavioral activation, and automatic thoughts were large [35]. Because alliance effects for IPIs have been attributed to participant relationships with the IPI programs in some studies [19,28], this analysis quantifies the frequency of responses related to each for comparison.

The findings bear on IPI treatment design, future directions for IPI alliance research, and the training of support personnel. As qualitative analysis is an inductive process, a priori hypotheses were not made.

### Terminology

*IPIs* are used in this manuscript as an umbrella term for mental health interventions that are wholly or partly administered through technology such as the internet or other electronic means. The use of IPIs throughout this paper is intended to refer to the broader literature of electronic-delivered interventions and to acknowledge that alliance is a pantheoretical construct that is present to greater or lesser degrees between users of IPIs and the program itself and the support person. We will use more specific terminology such as iCBT when referring to the data from this study and when relevant in reference to specific cited studies.

## Methods

### Participants

Participants in this study were a subset of those included in the program evaluation for MMB for veteran mothers [35]. Of the final sample (N=200) from the program evaluation, 184 (92%) veterans provided at least 1 response to the 4 open-ended questions and were therefore included in this analysis. A total of 758 individual responses were provided.

### Inclusion and Exclusion Criteria

The study sample consisted of veteran women enrolled in the MMB program who had completed a follow-up assessment at the 3-month postenrollment time point and had provided responses to at least 1 of the qualitative questions. Participants were excluded from the analysis if they did not provide a response to any of the 4 qualitative questions (16/200, 8%). Individual responses (10/758, 1.3%) were excluded if they were uninterpretable or ambiguous due to indecipherable words or uninterpretable brevity.

### Procedure

Participants for the larger MMB study were identified nationwide through the Veterans Affairs database of veterans whose private obstetrical care was paid for by the Veterans Affairs. Full recruitment and enrollment descriptions can be found in the initial program evaluation [35]. Participants were sent a follow-up questionnaire 3 months after the initial screening and enrollment in the MMB program, and the questionnaire included self-rated outcomes measures and 4 open-ended questions about the program, for which participants had unlimited characters to comment. Participants were free to skip questions that they did not wish to answer. Several participants opted not to answer any of the qualitative questions, whereas others answered only select questions. All the responses for the cohort included in the program analysis paper were included in this analysis [35]. The questions are referenced throughout the manuscript using the headings *program helpful*, *program improve*, *coach helpful*, and *coach improve* and are as follows:

1. Program helpful: “In what ways did you find the MomMoodBooster most helpful?”
2. Program improve: “How do you think the MomMoodBooster could have been improved?”
3. Coach helpful: “In what ways did you find the personal coach calls to be helpful?”
4. Coach improve: “How do you think the personal coach calls could have been improved?”

### Analysis

A modified iterative thematic analysis approach was used for data analysis. Coders used a consensual qualitative approach to the coding process and tallied themes to indicate the relative representation within the thematic structure. The six stages of thematic analysis delineated by Rennie [36] are as follows: (1) familiarization with the data via deep emersion, (2) systematic coding with collation of similar codes, (3) deriving themes and gathering relevant codes, (4) reviewing themes and checking for fit across individual codes and entire data set, (5) defining themes and ongoing refinement, and (6) producing the report. The first 2 authors independently assigned codes to each datum and then met regularly for an iterative process of reaching a consensus. Once a consensus was reached, an internal auditor reviewed the codes and provided feedback. The coders then reviewed the auditor comments until consensus was reached again, wherein the 2 coders resumed independent coding of domains and themes before meeting again to build consensus. The processes of internal audit and subsequent review and revision were undertaken at this stage as well.

### Positionality

The first author identifies as a mother who experienced postpartum depression and is a coach for the MMB program with 6 years of experience, including working with 105 participants in this sample. In addition, the first author has significant clinical experience working with women in the postpartum period. The second author identifies as a preparenthood man who functioned as the primary recruiter and contact person for the participants and was responsible for data management. The first and second authors acknowledged their experiences and power differences and worked to mitigate the effect of these factors during the coding process. The third author, who functioned as the auditor for the coding process, identifies as a woman without direct experience of motherhood or postpartum depression. This author, however, is familiar with the structure and content of the program in her role as a phone coach, commencing after this sample was collected. The fourth author identifies as a woman and is the principal investigator of this project. The fifth author identifies as a man and was the creator of the larger MMB for veteran women research project. He has worked in the area of perinatal depression research for 42 years. The fourth and fifth authors were involved in manuscript review and did not participate in data analysis. As a team, all authors are invested in women’s and veteran’s mental health and as such are most interested in how to improve the services provided through the MMB program.

Given the coders’ close ties to the program and direct experience with participants, the research team anticipated that relational aspects of the coaching interactions would emerge as important in the data. In order to check their biases related to the importance of the relationship, ambiguous codes referencing the coach were not coded as interpersonal unless the participant directly named their specific coach; however, they may have eventually been subsumed under interpersonal during the process of distilling down to themes. Coders worked hard to challenge each other with respect to the interpretation of interpersonal themes.

### Trustworthiness

The research team took several steps to attend to trustworthiness. The first was identifying positionality [37] to be transparent about their connection to the study participants and potential biases. Second, the team used an internal auditor and the process of consensus [38] throughout all the stages of coding and assigning to themes and domains. Third, the team journaled about their process during coding to document thought processes, challenges, and decision points. These have been included in the general audit trial [37]. Finally, an external auditor, a qualitative method expert who has no affiliation with the MMB program performed an audit of the coding process and randomly selected codes, the final thematic structure, and overall adherence to trustworthiness. No concerns were noted or changes were suggested.

### Ethics Approval

This study was approved by the University of Iowa Institutional Review Board (201310766 [IRB-03] and 201603779 [IRB-01]).

## Results

### Overview

The final sample consisted of 184 veterans who provided at least 1 qualitative response. Responses to the 4 questions from these participants were coded into a total of 748 codes due to multiple codes being assigned to some responses. Of the 748 total codes, 390 (52.1%) were program related and 358 (47.9%) were coach related. The 184 participants answered the 4 questions that are referred by the following headings: program helpful (n=176, 95.7%); program improve (n=142, 77.2%); coach helpful (n=162, 88%); and coach improve (n=118, 64.1%).

The thematic analysis resulted in a final structure consisting of five domains: (1) program accessibility and functionality, (2) content, (3) coaching, (4) change processes, and (5) barriers.

Table 1 delineates the thematic structure by including frequencies for each domain, and Multimedia Appendix 1 displays the thematic structure with definitions and example quotes. Quotes from participants have been numbered in the order they appear in this manuscript to further protect anonymity. Each participant number represents a unique individual, with relevant quotes provided by a wide range of participants.

**Table 1:** Relative representation of codes.

Domains, themes, and subthemes						Relative frequency, n (%)^a^
**Program accessibility and functionality**						
	**All program-related codes (n=390)**					
		Positive		17 (4.4)		
		**Improve (n=55)**		55 (14.1)		
			Access	13 (23.6)		
			Delivery mode	16 (29.1)		
			Functionality	11 (20)		
			Technical	15 (27.3)		
**Content (n=390)**						
	Positive				31 (7.9)	
	Improve				61 (15.6)	
	Specific helpful content				45 (11.5)	
**Coaching**						
	**All coach-related codes (n=358)**					
		General positives		36 (10.1)		
		**Amount (n=34)**		34 (9.5)		
			Increase	26 (76.5)		
			Did not want, did not receive, or got no benefit	6 (17.6)		
			Decrease	2 (5.9)		
**Change processes**						
	**Program (n=390)**					
		Intrapersonal		108 (27.7)		
		Coach		34 (8.7)		
		Accountability		3 (0.8)		
	**Coach (n=358)**					
		Intrapersonal		41 (11.5)		
		Relationship or interpersonal		98 (27.4)		
		Qualities		69 (19.3)		
		Practical assistance		32 (8.9)		
		Accountability		33 (9.2)		
**Barriers (n=748)**						
	Time				12 (1.6)	
	Motivation and mood				3 (0.4)	
	Contextual factors				7 (0.9)	
	Scheduling or rescheduling problems				16 (2.1)	
	Coaching delivery method				6 (0.8)	

^a^Percentages represent the relative number of responses for each theme or subtheme with the respective sample.

### Domain 1: Program Accessibility or Functionality

The *program accessibility/functionality* domain refers to comments addressing how participants accessed or would like to have accessed the content and how it functioned from a technical standpoint. This domain includes two themes: (1) *positive* (17/390, 4.4% of the program-related codes), which refers to nonspecific positive comments such as the one shared by participant 1: “Convenient, self-paced”; and (2) *improve* (55/390, 14.4% of the program-related codes). The *improve* theme captures four subthemes: (1) *access*, (2) *delivery mode*, (3) *functionality*, and (4) *technical*. The subtheme *access*, which represented 24% (13/55) of the *improve* codes, refers to recommendations related to the timing or duration of access to the program. For example, participant 2 suggested, “given at discharge from the hospital.” The subtheme *delivery mode*, which represented 29% (16/55) of the *improve* codes, refers to alternative modes of delivery that participants felt would have been beneficial such as those noted by participant 3: “Offered in a classroom setting.” The *functionality* subtheme, which represented 20% (11/55) of the *improve* codes, identified electronic formatting that would have facilitated greater engagement; for example, participant 4 suggested the program be, “More game like, not literature.” Finally, the *technical* subtheme, which represented 27% (15/55) of the *improve* codes, identified technological issues that affected participant experience such as the one commented by participant 5: “I had issues with inputting my daily mood.”

### Domain 2: Content

The *content* domain refers to comments identifying specific content from the program or more general comments made under the *program helpful and program improve* questions. This domain includes three themes: (1) *positive*, (2) *improve*, and (3) *specific helpful content*. *Positive,* referring to positive comments that were specific to content, accounted for 7.9% (31/390) of the program-related codes; for example, participant 6 noted, “I enjoyed the interactive [aspects].” *Improve,* accounting for 15.6% (61/390) of the program-related codes, refers to specific suggestions for ways the content could have better met participant needs such as the one requested by participant 7: “Make the program more diverse.” The *specific helpful content* subtheme, representing 11.5% (45/390) of the program-specific codes, refers to codes in which participants named specific pieces of content that they appreciated; for example, participant 8 noted, “Identifying negative thoughts and downward mood spiral.”

### Domain 3: Coaching

The *coaching* domain refers to general comments relating to the coach or the amount of coaching from any of the 4 questions. This domain consists of two themes: (1) *general positives*, accounting for 10.1% (36/358) of the coach-related codes, and (2) *amount*, accounting for 9.5% (34/358) of the coach-related codes. The codes under the theme *general positives* were nonspecific satisfaction comments such as the one commented by participant 9: “No improvements needed.” Three subthemes emerged under the *amount* theme: (1) the *increase* subtheme, which accounted for 77% (26/34) of the codes, was exemplified by participant 10, who expressed, “Continuation a week or two after the program ended,” and participant 11, who shared, “More phone calls”; (2) the *didn’t want, didn’t receive, or got no benefit* subtheme was less common, representing only 18% (6/34) of the *amount* codes; for example, participant 12 expressed, “What calls?? Received none,” and participant 13 stated, “I never spoke with anyone, that is not what I wanted from the program”; and (3) the *decrease* subtheme was the least common, representing only 6% (2/34) of the *amount* codes; for example, participant 14 stated, “More online activities, less calls.” (see Figure S1 in Multimedia Appendix 2 for visual depiction).

### Domain 4: Change Processes

The fourth domain, *change processes*, refers to specific intrapersonal, relational, and practical influences that participants identified. These are captured by two themes: (1) *program* and (2) *coach*, with 3 and 5 subthemes, respectively. The *program* theme refers to codes that identified change processes either specifically related to program content or nonspecifically under the *program helpful* question. The *coach* theme refers to codes that specifically indicated a link to coaching or were responses under the *coach helpful* or *coach improve* questions. Replicated subthemes (interpersonal and accountability) across *program* and *coach* themes emerged through the inductive coding process. Coding these subthemes separately, rather than collapsing, was prioritized to compare the relative contributions across themes (see Figure S2 in Multimedia Appendix 2 for visual depiction).

Under the *program* theme, the first subtheme, *intrapersonal*, refers to the internal process of personal growth and change identified by the participants. Of the 390 program-related codes, 108 (27.7%) codes were included under this subtheme. These included codes were managing negative thoughts, increased self-awareness, learning coping strategies, increased agency, and others. Participant 15 commented, “I learned how to be calm and take care of my baby.” Some participants indicated that the coach was the most helpful aspect of the program; for example, in response to the *program helpful* question, participant 16 commented, “Helpful to talk to someone who is supporting.” Of the 390 program-related codes, 34 (8.7%) codes referenced the coach and were therefore included as a subtheme of the program-related change processes. These were coded separately from the *coach* theme for 2 reasons. First, these comments were more general than those captured under the *coach* theme and second, because it felt important to differentiate and identify how often the coach was named as the salient helpful aspect of the program as a whole. The third subtheme under *program* theme was *accountability*. This subtheme refers to comments indicating that being held accountable was the most helpful aspect of the program. Only 3 respondents identified *accountability* in response to the *program helpful* question, and it represented 0.8% (3/390) of the program-related codes. Interestingly, 2 of these participants also named relationship factors in other responses; for example, participant 17 who commented under *program helpful*, “Having a system to be accountable to,” which was coded as *accountability*, also commented under *coach helpful* “having a live person to check in with is very comforting,” which was coded as *relationship/interpersonal* (which are subthemes under *coach* theme).

The second theme under the *change processes* domain is *coach,* which included 5 subthemes. *Intrapersonal* (41/358, 11.5% of the coach-related codes) refers to growth and change identified by the participant specifically credited to the coaching interaction. Some of the intrapersonal processes include self-reflection, challenging negative thinking, perspective-taking, increased self-compassion, normalizing, and processing problems. For example, participant 18 commented, “Helped me reflect on myself and identify negative talk to myself.” (see Figure S3 in Multimedia Appendix 2 for visual depiction).

The second *coach* subtheme, *relationship/interpersonal* (98/358, 27.4%), refers to codes that referenced the importance of the relationship between the participant and the coach. Some of the codes in this domain were requests from participants for what they would like to be different in the coaching interactions such as the one shared by participant 19: “more input, deeper conversation,” which was coded as *increase depth*. Others were experienced by the participant; for example, participant 20 shared “[specific coach name] listens even if I haven’t completed the week and gives praise and good thoughts,” which was coded as *relationship* because of the use of the coach’s name and *coach qualities, active listener and supportive*. Participant 21 shared, “I really liked my coach” which was coded as *interpersonal relationship*.

The third *coach* subtheme, *qualities*, represented 19.3% (69/358) of the coach-related codes and refers to specific attributes named by participants. For example, the comment by participant 22, “Felt very genuine,” was coded as *genuine*, and the comment by participant 23, “No matter what was going on in my life she always empathized and worked with me,” was coded as *empathetic*. Note that this response, which is similar to the one shared by participant 20 above, was not coded as *relationship*, as the participant did not name her specific coach (see Figure S4 in Multimedia Appendix 2 for visual depiction).

The fourth *coach* subtheme, *practical assistance,* refers to codes that identified specific practical ways that coaches helped participants such as assistance with technical difficulties and clarification of content. This subtheme accounted for 8.9% (32/358) of the coach-related codes.

The fifth *coach* subtheme, *accountability*, refers to codes that specifically identified the coach as the agent of holding participants accountable. These codes represented 9.2% (33/358) of the coach-related codes. Interestingly, 24% (8/33) of the participants who provided responses that were coded under *coach* *accountability* subtheme also provided responses that were coded under *relationship/interpersonal* subtheme.

### Domain 5: Barriers

The final domain refers to the challenges that respondents named that prevented them from engaging with the content and coaching. Five themes were captured under *barriers* including (1) time, (2) motivation and mood, (3) coaching delivery method, (4) contextual factors, and (5) scheduling and rescheduling problems (representing n=12, 1.6%; n=3, 0.4%; n=7, 0.9%; n=16, 2.1%; and n=6, 0.8%; respectively, of all 748 codes). Table 1 provides the relative frequency of the themes under *barrier* domain, and Multimedia Appendix 1 provides descriptions and selected quotes for each of these themes.

## Discussion

This study aimed to illuminate participant perspectives regarding engagement with a telephone-supported IPI for postpartum depression, the iCBT MMB program. Results of this study will inform future IPI alliance research, training of support personnel, program development, and allocation of resources for supported IPIs.

### Principal Findings

Our thematic analysis of 184 veterans’ responses to 4 open-ended questions regarding MMB program content and impressions of the support person provided a unique look at participant perspectives of this supported iCBT. Furthermore, we were able to compare participant perspectives across program content and the support person specifically. Overall, 3 of the 5 identified domains, *program content, accessibility/functionality*, and *barriers*, provide ideas for program developers regarding particularly helpful aspects of program content and design. In addition, some of the information found in the *intrapersonal* theme under *change processes* domain may identify specific processes that can be targeted via content in future human-supported programs.

More significantly, these data illuminate some of the important change processes facilitated by the support relationship and specific qualities of the support person that elicit participant responses. For example, the intrapersonal subthemes and relationship or interpersonal subthemes represent the largest proportion of codes. This indicates that interpersonal processes and relationship factors may warrant further investigation in the context of alliance-based research for iCBT specifically and IPIs more generally. Although some program-related intrapersonal processes are clearly attributable to the program content (eg, video content that served a normalizing purpose), others may be attributed to either the content or the coach. Without a clear reference to one or the other, such processes were assigned to the content. However, given the number of participants who referred to the coach under the *program helpful* question, it is clear that some were considering the entirety of the program, rather than strictly the content, when referencing intrapersonal processes. Future IPI alliance research may want to be mindful of construct validity issues and whether questionnaires probe intrapersonal or interpersonal processes. Similarly, qualitative researchers may want to be mindful of how they query these processes.

Our findings also suggest that the coach is perceived as one of the most important aspects of the program, as approximately 20% of the responses under *program helpful* referenced the coach. Furthermore, of the participants who identified the amount of time with the coach under *coach improve*, approximately 3 times as many indicated wanting more contacts compared with the combined total across *no change*, *didn’t need*, or *decrease* codes, suggesting that participants valued the support time and, in many cases, wished for more. Significantly, approximately 60% of the coach codes (from the *coach helpful* and *coach improve* questions) identified relationship or interpersonal factors, and >40% identified coach qualities that cannot exist outside of an interpersonal relationship. In summary, the participants acknowledged the importance of the coaching relationship. This is in line with previous research emphasizing the importance of the therapeutic alliance in supported IPIs [10,16,17].

In contrast, fewer participants identified accountability as an important aspect of the program or coaching compared with other themes under *change process* domain. *Accountability* was identified in <1% of the *program helpful* codes, and in response to the *coach helpful* question, *accountability* was identified in <20% of the codes. However, because *accountability* has been found to be related to better adherence to IPIs [39], it is important to consider this finding more closely. Previous findings have shown that clinician contact during iCBT has been positively associated with adherence [40] and that adherence is positively associated with outcomes [41], suggesting that greater doses of the intervention improve outcomes.

### Limitations

This study has several limitations that should be acknowledged. First, the 4 questions that were used to collect these data were not crafted with the intention of completing a qualitative analysis and were not designed for exploring the importance of the coach relationship in comparison with the program content. As a result, there is some ambiguity in the responses provided under the *program helpful* question. In addition, some of the emphases in the results on coaching and the relationship could be reflecting the specificity of one set of questions compared with the other. Second, participant responses were very brief in many cases, meaning that coders had to interpret meaning using familiarity with this sample and the program overall. Despite constantly challenging assumptions and biases, elements of bias may have certainly influenced the final coding and thematic structure. Finally, the final thematic structure does not represent the women who were noncompleters in the study or who did not provide follow-up data. It is possible that the nonresponders had very different experiences with the program and coaches that were not captured in this study.

### Comparison With Prior Work

One model of support, Supportive Accountability [42], a manualized model developed to address the low adherence rates found for unsupported iCBT [41], posits that reciprocity exists in the participant-coach relationship, and a strong therapeutic bond should enhance the effects of accountability [39]; however, the mechanism of the association between clinician contact and adherence is unclear. Therapeutic alliance is one possibility; however, previous findings suggest that the therapeutic alliance and adherence may contribute separately to outcomes. For instance, Bur et al [10] found that alliance and adherence mediated the effect of guidance; however, alliance contributed to better outcomes separately from adherence. This is consistent with the results of this analysis, which suggests that the focus of the support person is more importantly placed on alliance-building factors than on accountability and adherence by most participants.

It should be noted that accountability and alliance are not mutually exclusive, and one should not be used at the expense of the other. Our findings suggest that being responsive to client needs in IPIs is likely as important as it is in FTF therapy [43], and support should be approached from a client-centered perspective. Although some researchers have found that standardized feedback was as effective as individualized guidance [10], participants in this study, by contrast, identified the importance of experiencing the program as responsive and named ways by which a supported IPI could meet individual needs. One of these is to understand the individual needs for accountability, as seemingly few participants are motivated by accountability alone. Other program-based responsiveness strategies identified by these results includes maximizing access options, representing diverse identities, including social components, and providing interactive components. Other support-based responsiveness strategies included translating tools into real life, deepening the conversations, and focusing on the unique dyadic relationship and unique needs of program users.

Inherent in the adherence literature is the idea that specific ingredients of the IPI or iCBT content are responsible for outcomes, and higher “doses” of these ingredients are associated with more positive outcomes. In contrast to this medical model of therapeutic change is the contextual model [15], which emphasizes therapeutic alliance. Within the IPI literature, the alliance has thus far been defined in terms of task, goal, and bond subscales given by Bordin [12,13]. The present results suggest that more recent work on therapeutic alliance may be a better fit in the case of IPIs. For example, in the tripartite model of the therapeutic relationship (ie, the real relationship, the working alliance, and the transference configuration) given by Gelso [44], the real relationship is defined as “the personal relationship between therapist and patient marked by the extent to which each is genuine with the other” [44] and perceives and experiences the other in (realistic) ways. The results of this study appear to reflect this definition, as participants identified coach genuineness as an important aspect of their experience and expressed the desire for the coaching interactions to be personal and deeper than is possible with standardized support or an emphasis on accountability to completing content.

Further refinement of the model given by Gelso [44] has resulted in the tripartite model of the real relationship composed of belongingness, empathy, and expectations [45]. Belongingness, in this framework, references “Attachment Theory” by Bowlby and Base [46] and as such is proposed to influence positive outcomes through the effects of a participant feeling connected to another person who cares about their well-being. This is consistent with the suggestion by Bur et al [10] that it is not the actual contact itself that increases alliance but that the participant knows that a real person will support them during treatment. Our data appear to align with this idea as codes emerged such as feeling connected, human connection, and feeling supported, even when total time in contact with the coach was relatively low. However, the preponderance of codes indicating relationship factors suggests that additional work will be needed to further define the role of attachment and relationship factors in supported IPIs. The results of this study, therefore, provide future research directions, including more nuanced definitions of the alliance and the real relationship, that align more closely with the current literature.

Further refinement may also be necessary in terms of differentiating alliances based on the mode of contact. The meta-analysis by Kaiser et al [16] determined that there was no difference in outcomes based on the frequency of contact with the support person, mode of contact, or availability of self-help content. However, most of the studies included in the meta-analysis by Kaiser et al [16] used email as the mode of support. It may be that different forms of support elicit different reactions to the task, goal, and bond subscales of the WAI or that different alliance measures are needed for differently supported IPIs. The results of this study suggest that the bond subscale could emerge as more important for support provided synchronously by phone. In addition, other researchers have suggested that the relevance of the alliance for therapy outcomes could differ among different kinds of disorders and different client groups [17]. The emphasis on the relationship with the coach in this study could be a reflection of the specific population (ie, veteran women in the postpartum period) and the specific concern (ie, postpartum depression).

### Conclusions

These findings provide a unique perspective regarding participant experiences in a supported iCBT program for postpartum depression. These data suggest that the most helpful coaches are those who are empathetic and validating, supportive and nonjudgmental, flexible, patient, and genuine. These qualities are highly relevant for trainees in health service delivery and research. Similarly, coaches might avoid emphasizing accountability alone unless the participant specifically identifies this as their primary need from support. Several participants commented that they wanted their coaching sessions to feel personal, to include conversation beyond simply what was happening with program content, to deepen and explore emotions, and to feel that the coach was genuinely interested in how they were feeling. As such, coaches might work to flexibly apply program content to the participant’s ongoing experience and context while prioritizing responding with genuineness and empathy. Creators of manualized support models should consider how these results inform the training of their support personnel.

Future research may explore associations among qualities of coaches, amount of synchronous telephone contact, alliance ratings, and participant outcomes. Our findings demonstrate that the participants were capable of discerning beneficial coach qualities through an IPI program in which there was synchronous audio contact. Previous research has suggested that it is possible to form therapeutic alliance within 2 weeks of initiating an intervention and that early alliance associations with outcomes may indicate that guidance may not be necessary for the entire duration of the IPI [10]. This study’s data suggest that the users of iCBT supported by phone may desire more contact with support personnel and therefore engagement throughout their use of the IPI. Given that most participants in this sample wanted more coaching, in terms of frequency, length, or duration, program designers will want to carefully consider the balance between participants’ wishes, efficient resource allocation, and therapeutic benefit. These considerations may be specific to telephone-based support and will therefore need follow-up research to discern questions of timing and dose effects of telephone-based support.

These results call into question previous research that has indicated that the bond subscale of the model by Bordin [12,13] is less impactful to the alliance-outcome relationship than the task and goal subscales [10,18]. It may be that conceptualization of the alliance in IPIs needs to be further developed and that commonly used measures of the alliance, the WAI [30], need to be further studied for psychometric validity when used to assess alliance in IPIs.

Kaiser et al [16] call for attention to therapeutic alliance in the design of IPIs and specifically the elements that may promote alliance such as mode of communication, individualizing feedback, and elements that contribute to therapist credibility. Pihlaja et al [23], however, commented that detailed descriptions of therapist characteristics for iCBT were challenging and therefore beyond scientific scrutiny because of the range of probable predictors of alliance. The data of this study provide some information for how designers and alliance researchers might consider the support aspect of IPIs.

Although some have suggested that alliance-related factors may be less impactful in IPIs [22], the participant perspectives conveyed in these findings suggest otherwise and are consistent with more recent research. However, a large gap in the IPI literature is the provision of support from a theoretical perspective. The conceptualization of the real relationship within the tripartite model of the alliance by Gelso [44] and further expansion to the tripartite model of the real relationship by Budge and Wampold [45] may be fruitful directions for IPI researchers.

Important to consider in future research is the model from which coaches are trained and supervised, as this is likely to impact treatment outcomes, engagement, adherence, and attrition. Future research with these data is planned that will compare the association between relationship factors, engagement measures, and outcomes under accountability-focused and alliance-focused support models.

The literature on therapist responsiveness also suggests that strict standardization of support may be counterproductive. Swift et al [47] summarized that matching a treatment to client preferences, compared with not matching the treatment to the client preferences, increased success rates, led to more progress, and resulted in fewer dropouts. When a participant is engaging with the IPI content, an appropriate place to tailor treatment to the individual, via attending to preferences, is the support relationship. Our findings support therapist responsiveness to relationship factors as a particularly important component of treatment.

Although these participants engaged with their coaches for an average of 12 minutes per coaching session [35], much less than the typical FTF therapy, some of these findings may be alluding to the distinction between counseling therapy and supportive coaching. Future work is warranted to examine the extent to which human contact is perceived as adjunctive to IPIs and vice versa and for whom it is adjunctive.

Perhaps the most important feature in the training context was that the coaches for this program were doctoral level trainees in clinical and counseling psychology. Future implementations of iCBT for postpartum depression and other IPIs may use trainees knowing that the therapeutic alliance and responsiveness variables identified as beneficial herein are trainable and regarded highly by participants. However, given the nature of the valued coach characteristics identified herein, properly trained peers may also be a viable and scalable option for future IPIs and research.

One strength of this study is the relatively large sample size (n=184), particularly in terms of typical qualitative studies. Several characteristics of the sample suggest that the results are generalizable to other supported iCBT interventions and supported IPIs. For example, participant demographics were diverse and, through nationwide recruitment, represented diverse areas of the country, including both rural and urban locations. In addition, previous work demonstrated that engagement and outcomes of this program were comparable with those of other IPIs [35].

Given that most codes were not specific to CBT ingredients or to the functioning of this specific program, but instead were intrapersonal and process comments, these results are likely generalizable to non-CBT IPIs. Furthermore, qualitative analysis, as a methodology, is nearly absent from the IPI literature, and as such, these data provide a unique perspective into potential mechanisms of change, which has been an elusive aspect of process and outcomes research [48].

## Appendix

Multimedia Appendix 1 Complete thematic structure including definitions and participant quotes.

Multimedia Appendix 2 Relative frequency of codes indicating amount of coaching preferences, relative contributions to change processes by program- and coach-related codes, relative representation of change processes attributed to coach, and relative representation of coach qualities.

## Abbreviations

CBT: cognitive behavioral therapy
FTF: face-to-face
iCBT: internet-delivered cognitive behavioral therapy
IPI: internet-delivered psychological intervention
MMB: MomMoodBooster
WAI: Working Alliance Inventory
