# Supplementary material for: Relationship Factors in Internet-Delivered Psychological Interventions for Veterans Experiencing Postpartum Depression: Qualitative Analysis
Source: JMIR Ment Health. 2023 Aug 15;10:e46061. doi: 10.2196/46061 (PMC10466152; doi:10.2196/46061)
Supplement: Multimedia Appendix 1 [file mental_v10i1e46061_app1.docx]

**Appendix**

*Thematic structure of domains, themes, and sub-themes, including definitions.*

| Domain | Theme | Sub-theme | Definition |
| --- | --- | --- | --- |
| Program accessibility/ functionality | | | Comments referring to how participants access the program or how it functions |
|  | Positive |  | Non-specific positive response including those indicating that the program was convenient and easy to use, as well as appreciating the self-paced nature.  #26, “Easy to use online.”  #27, “I liked the online program.” |
|  | Improve |  |  |
|  |  | Access | Comments addressing when and how long participants would have liked to have had the program available. These include being offered at hospital discharged, having flexible and indefinite access to program materials, and for all content to open immediately instead of sequentially.  #28, “Keep available online to previous users indefinitely.”  #29, “stay open always.” |
|  |  | Delivery Mode | Comments identifying how participants would like to have the program be available. These include mobile-capable, delivered by video, and content delivered in person.  #30, “Mobile app! Easier for busy moms.”  #31, “ Maybe video conference.” |
|  |  | Functionality | Refers to comments identifying aspects of the electronic formatting that would improve the program. These include up-to-date web formatting, making the program more game-like/interactive, having an automatic connection between the mood tracking and pleasant activities tracking, having a participant forum, and indefinite character capabilities for content capture interactions which would allow more personalization.  #32, “I think we should be able to journal more but it limited character count.”  #33, “Felt repetitive, change up the structure.” |
|  |  | Technical | Refers to both technical problems experienced by some participants such as web-site glitches and log-on troubles, as well as suggestions for additional technical capabilities. These include automated reminders to log on and to practice self-care, forced mood tracking, and automatic connection between pleasant activities and mood tracking as a means of providing insight.  #34, “fix the website, I wasn’t able to log in.”  #35, “[Provide] reminders to log in.” |
| Content |  |  | Comments relating to content of the program either through reference to specific aspects of the program material or more general comments under the program helpful and program improve questions. |
|  | Positive |  | Refers to non-specific positives about the program. For example, participant.  #24, “The content was great.”  #25, “Relevant information.” |
|  | Improve |  | Refers to comments indicating that the quality of the information could be improved and/or deepened, that social components would be helpful, and that more diverse identities should be represented such as young moms, single moms, moms with deployed partners, and moms with co-occurring mental health concerns.  #36, “Better videos and imagery.”  #37, “New slides with younger moms.” |
|  | Specific Helpful Content |  | Captures specific content participants identified as having been helpful. These include the downward mood spiral, the practice activities in general, pleasant activity tracking and more.  #38, “CBT training – spiral thinking.”  #39, “It gave me useful, thoughtful methods to lift my mood.” |
| Coaching |  |  | Codes relating to the coach from any of the four questions |
|  | General Positives |  | Non-specific positive comments about the coaching.  #40, “She did great.”  #41, “Very nice lady, easy to talk to.” |
|  | Amount |  | Codes referring to the amount of coaching received |
|  |  | Increase | Codes referring to wanting more time with the coach, either more contact each week, longer calls, or longer duration of access to the coaching.  #42, “Longer time or more calls.”  #43, “If they were one hour long.” |
|  |  | Did not want, did not receive, or got no benefit | Codes referring to having experienced troubles connecting with the coach, found no benefit from the time with the coach, or were not wanting to engage in coaching as part of their experience.  #44, “didn’t have any.”  #45, “I could imagine they were; did not get any.” |
|  |  | Decrease | Codes referring to wanting to engage with the coach but less often or for less time than what the participant experienced.  #46, “Sometimes I felt uncomfortable saying things out loud so maybe have a written communication and calls every other week.  #47, “N/A – I never spoke with anyone. That is not what I want from the program.” |
| Change processes |  |  | Codes identifying specific intra-personal change process or naming factors contributing to change |
|  | Program |  |  |
|  |  | Intra-personal | Codes indicating that the program facilitated change processes such as managing negative thinking, increasing self-reflection and self-awareness, normalizing experience, and learning coping strategies such as managing mood, behavioral activation, and self-care.  #15, “I learned how to be calm and take care of my baby.”  #48, “Made me aware of my thoughts and feelings.” |
|  |  | Coach | General statements indicating that the coach was the most helpful part of the program, shared in response to the Program Helpful question.  #16, “Helpful to talk to someone who is supporting.”  #49, “Having some to call and talk with.” |
|  |  | Accountability | Codes indicating that the program provided a structure that held them accountable.  #50, “Having a system to be accountable to.” |
|  | Coach |  |  |
|  |  | Intra-personal | Statements referencing the coach’s role in facilitating intra-personal change.  #18, “Weekly calls with my coach really helped me reflect on myself and identify negative talk to myself.”  #51, “Helped me be less critical of myself.” |
|  |  | Relationship/Inter-personal | Codes indicating that the relationship with the coach was important.  #52, “[coach name] was very helpful and encouraging, I miss talking with her.”  #53, “She was personable.” |
|  |  | Qualities | Codes naming specific qualities either hoped for or received from coach such as empathy, validation, support, kindness etc.  #54, “Felt very genuine”  #55, “It was nice to talk with someone who understood and validated my feelings.” |
|  |  | Practical Assistance | Participants referencing assistance of the coach helping with technical troubles, or clarification of content.  #56, “Clarification.” |
|  |  | Accountability | Codes referencing the coach as helping participant remain accountable (Note: 8 of these also referenced the relationship).  #17 who commented under Program Helpful, “Having a system to be accountable to.”  #57, “Held me accountable to stay on schedule.” |
| Barriers |  | Time | Codes referring to time related barriers such as work and multi-tasking demands.  #58, “I have not found the time to even start the program.”  #59, “It is hard to find time.” |
|  |  | Motivation | Codes referencing challenges with being motivated to engage with the program.  #60, “I was not very motivated to do it.”  #61, “It’s really hard to talk in the phone or get online some days.” |
|  |  | Contextual factors | Codes referring to contextual barriers such as computer problems or noise of kids interfering with phone calls.  #61, “It is really hard to talk on the phone, too much noise and commotion from kids, really frustrating.”  #62, “No internet.” |
|  |  | (re)Scheduling problems | Codes referring to challenging with scheduling or reaching coaches for rescheduling missed calls.  #63, “Easier scheduling.”  #64, “Scheduling with coach.” |
|  |  | Coaching Delivery Method | Codes referring lack of engagement due to wanting a different form of coaching such as in-person or video chat.  #65, “May have felt more personal as video calls.”  #66, “Maybe changing to in-person/Skype, for face-to-face.” |

*Note*. This table summarizes and defines the domains, themes, and subthemes with representative quotes.
