# Supplementary material for: Relationship Factors in Internet-Delivered Psychological Interventions for Veterans Experiencing Postpartum Depression: Qualitative Analysis
Source: JMIR Ment Health. 2023 Aug 15;10:e46061. doi: 10.2196/46061 (PMC10466152; doi:10.2196/46061)

Figure S1. Relative frequency of codes indicating amount of coaching preferences. Percentages are based on 34 codes referencing amount of coaching.


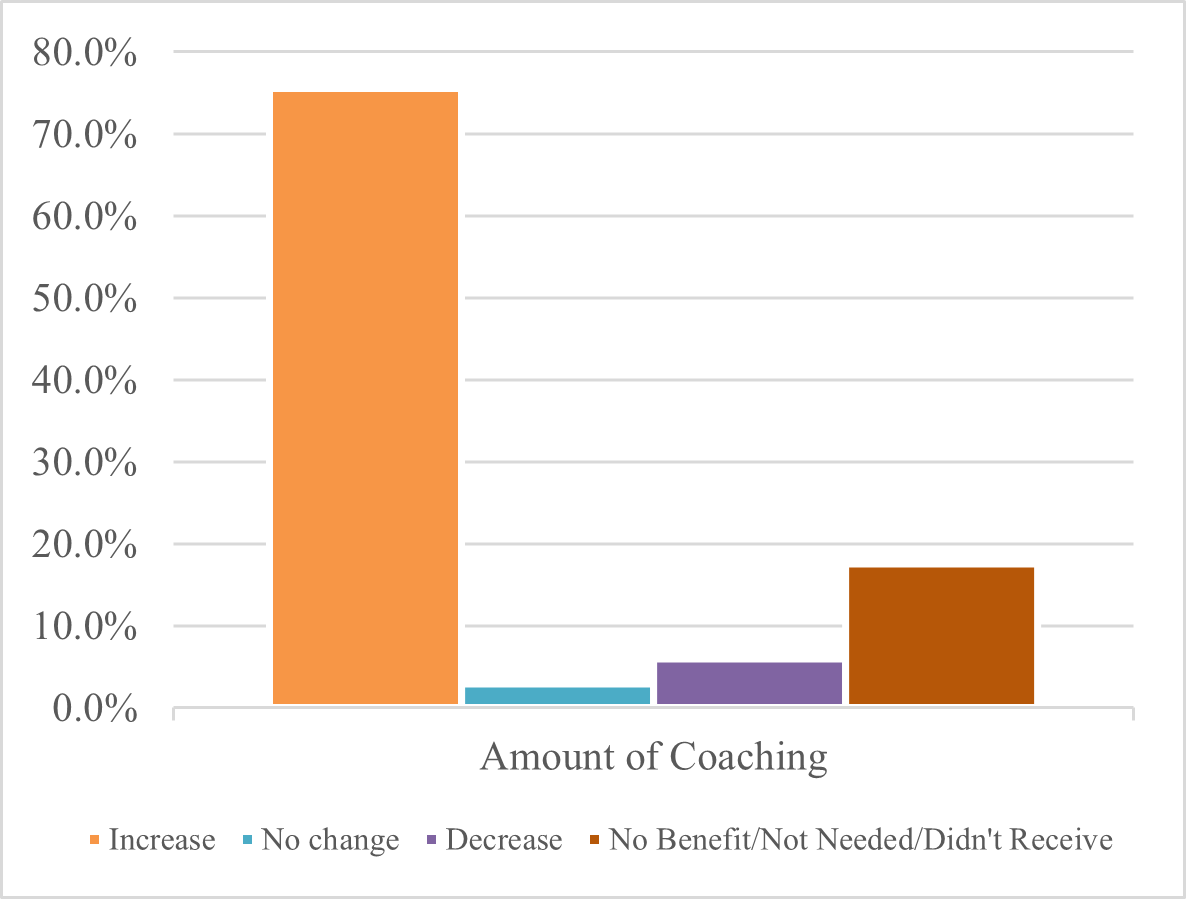


Figure S2. Relative contributions to change processes by program- and coach-related codes. Relative contributions are represented by the number of codes under each theme.


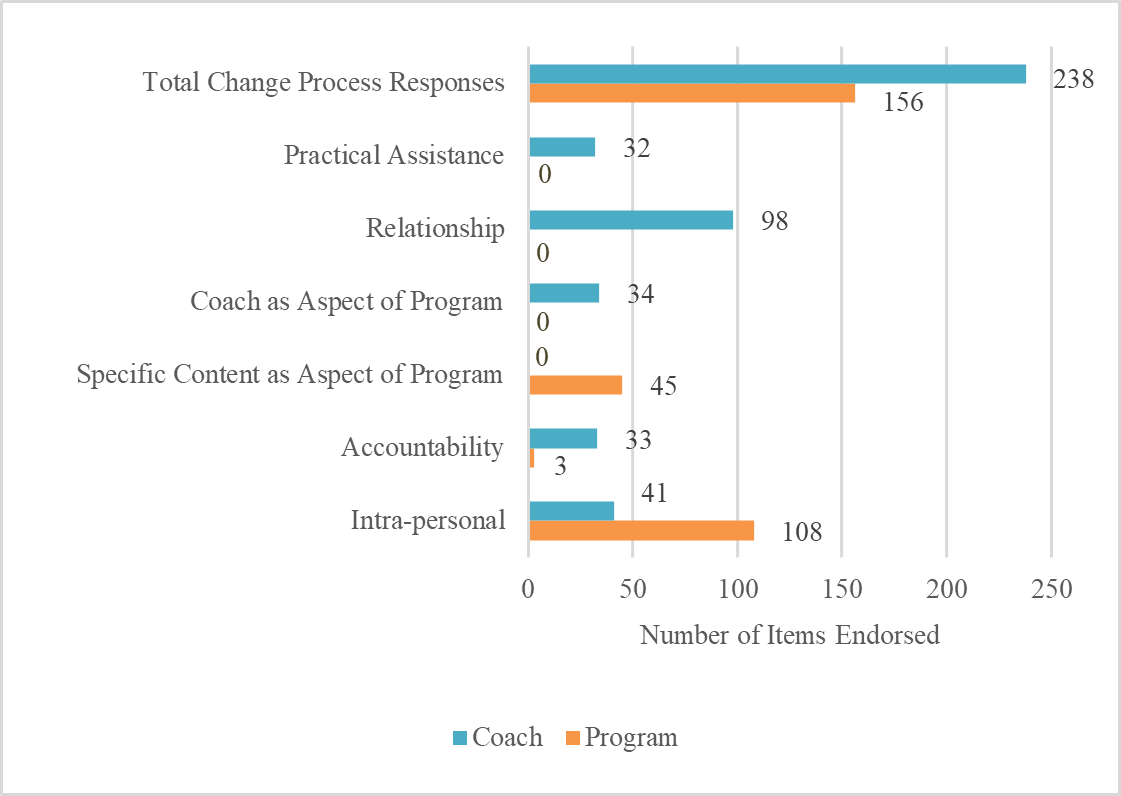


Figure S3. Relative representation of change processes attributed to coach. Change processes are represented by the number of codes captured in each category.


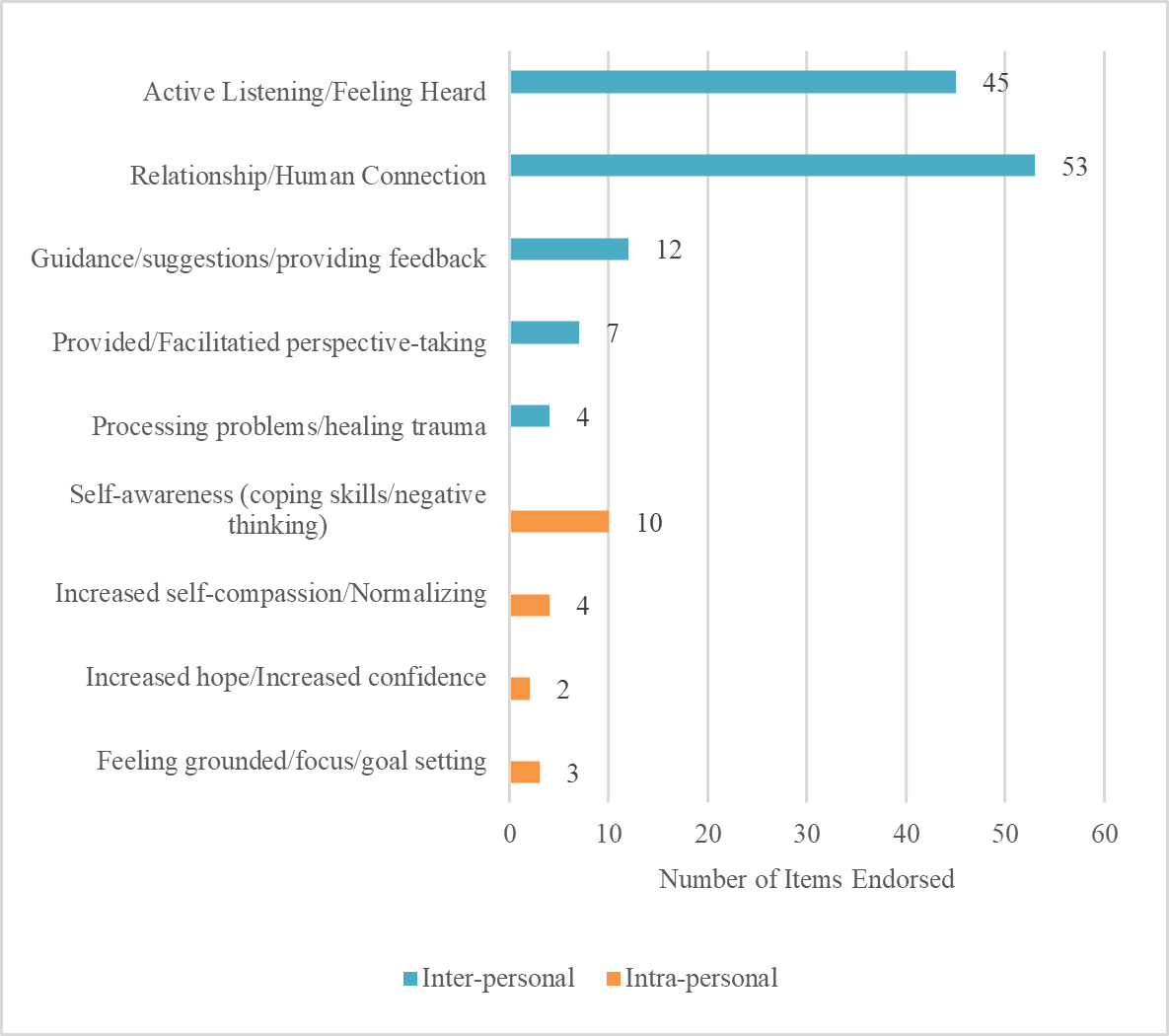


Figure S4. Relative representation of coach qualities. Coach quality is represented by the number of codes captured in each category.


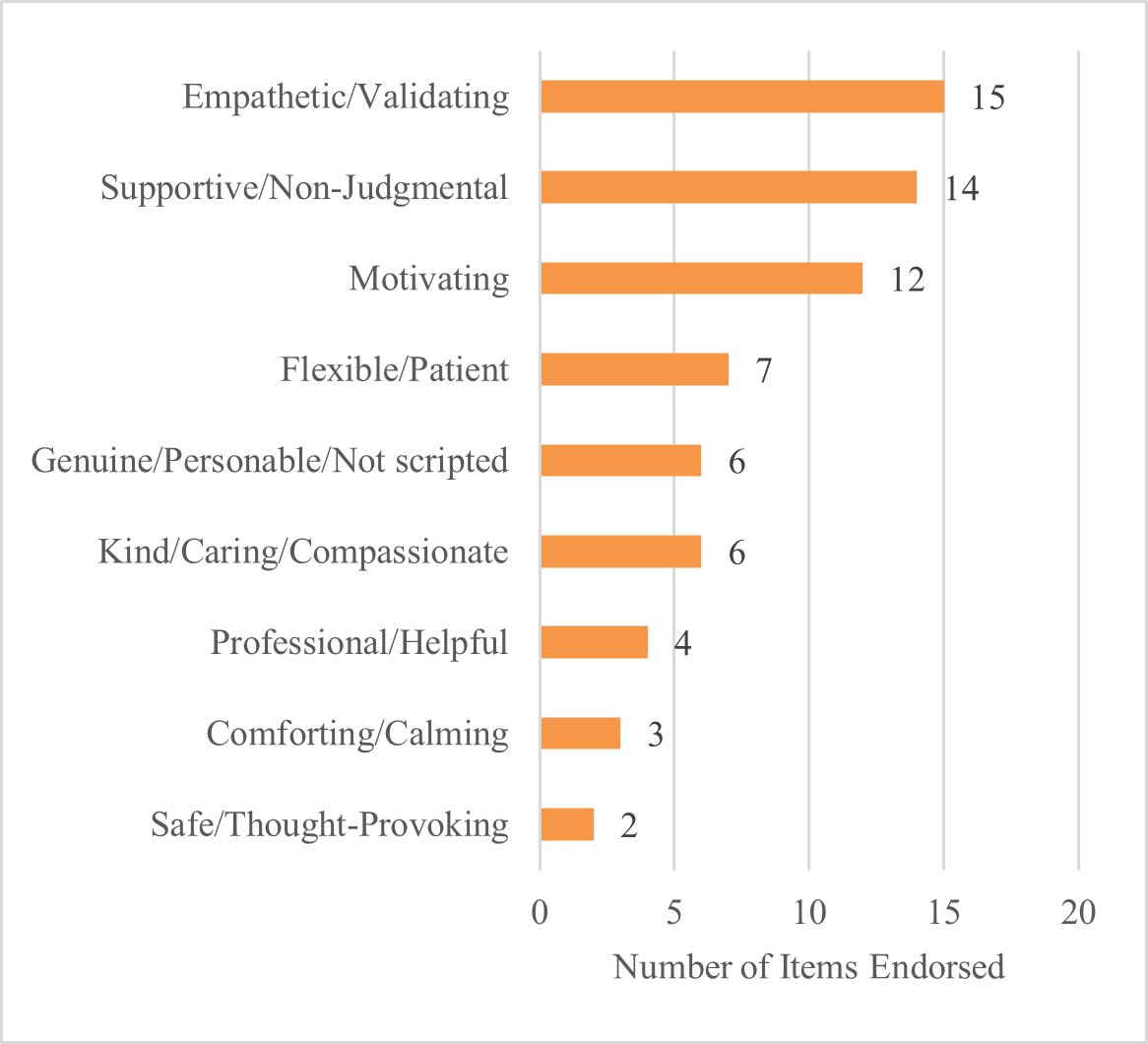

Supplement: Multimedia Appendix 2 [file mental_v10i1e46061_app2.docx]
